# Correction to "DetectEV: a Functional Enzymatic Assay to Assess Integrity and Bioactivity of Extracellular Vesicles"

**DOI:** 10.1002/jev2.70075

**Published:** 2025-04-16

1

G. Adamo, S. Picciotto, P. Gargano, et al, “DetectEV: A Functional Enzymatic Assay to Assess Integrity and Bioactivity of Extracellular Vesicles,” *Journal of Extracellular Vesicles*, 14 (2025): e70030. https://doi.org/10.1002/jev2.70030

In the originally published article, the term ‘mol’ (along with its derivatives ‘nmol’ and ‘µmol’) was mistakenly used instead of ‘M’ (molarity). This issue affects only the notation and does not impact the data, observations, or conclusions of the article. The correct notation is ‘M’ (along with its derivatives ‘nM’ and ‘µM’), as the reference is to concentrations rather than absolute quantities.

We apologise for this error.

